# Association of Neuropsychiatric Symptoms and General Psychopathology with Markers of Neurodegeneration in the General Population

**DOI:** 10.1002/alz.092340

**Published:** 2025-01-03

**Authors:** Rika Etteldorf, Folgerdiena M. de Vries, Ulrich Ettinger, Monique M.B. Breteler

**Affiliations:** ^1^ Population Health Sciences, German Center for Neurodegenerative Diseases (DZNE), Bonn Germany; ^2^ University of Bonn, Bonn Germany; ^3^ Institute for Medical Biometry, Informatics and Epidemiology (IMBIE), Faculty of Medicine, University of Bonn, Bonn Germany

## Abstract

**Background:**

Mental disorders have been linked to an increased risk of developing dementia including Alzheimer’s disease. However, previous studies have typically focused on individuals who meet diagnostic criteria for a given psychiatric disorder. The goal of our study was to examine whether diverse neuropsychiatric symptoms are related to early markers of neurodegeneration in the general population.

**Method:**

The analysis was based on 8,318 participants (aged 30‐95) from the population‐based Rhineland Study. Neuropsychiatric symptoms (perceived stress, depressive symptoms, anxiety symptoms, attention deficit hyperactivity (ADHD) symptoms, obsessive‐compulsive disorder (OCD) symptoms) were measured using self‐report questionnaires. A general psychopathology factor was generated using principal component analysis (PCA). Cognitive performance was measured across different cognitive domains. Whole‐brain and regional brain volumes as well as white matter hyperintensities (WMH) were obtained from magnetic resonance images (3T Siemens MAGNETOM Prisma). Plasma neurofilament light chain (NfL) levels were assessed using Quanterix’s Simoa NF‐light™ assay. Relations of symptom scores with markers of neurodegeneration were quantified using multivariable linear regression models. We performed a sensitivity analysis by excluding participants reporting a neurodegenerative or psychiatric disease (n = 1,784).

**Result:**

Participants with more severe neuropsychiatric symptoms and higher scores on the psychopathology factor performed significantly worse in all fluid cognitive domains (standardized beta coefficients (β) range between ‐0.01 and ‐0.06). The strength of association between neuropsychiatric symptoms and cognitive performance increased with age. Furthermore, neuropsychiatric symptoms (except for ADHD symptoms) and general psychopathology were associated with smaller grey matter volume (β between ‐0.00 and ‐0.02). Perceived stress, depressive symptoms, and anxiety were additionally associated with higher WMH load (β between 0.02 and 0.04), while OCD symptoms were associated with thinner cerebral cortex (β = ‐0.05 [‐0.07, ‐0.02]). Sensitivity analysis showed robustness of the associations. We found no associations between neuropsychiatric symptoms and NfL levels.

**Conclusion:**

Various neuropsychiatric symptoms and a generally higher level of psychopathology are associated with poorer cognitive performance, grey matter atrophy, and WMH burden, even in adults without a psychiatric diagnosis. However, we did not find direct evidence of neuroaxonal injury, as indicated by the absence of an association with NfL.

